# Preparation and Finite Element Analysis of Fly Ash/HDPE Composites for Large Diameter Bellows

**DOI:** 10.3390/polym13234204

**Published:** 2021-11-30

**Authors:** Angxuan Wu, Lan Jia, Wenwen Yu, Fengbo Zhu, Fuyong Liu, Yanqin Wang, Guoyun Lu, Shuhao Qin, Dongyang Gao, Hua Wang, Xiaogang Wu, Qiang Zheng

**Affiliations:** 1 College of Materials Science & Engineering, Taiyuan University of Technology, Taiyuan 030024, China; wuangxuan0203@link.tyut.edu.cn (A.W.); jialan@tyut.edu.cn (L.J.); yuwenwen@tyut.edu.cn (W.Y.); zhufengbo@tyut.edu.cn (F.Z.); liufuyong@tyut.edu.cn (F.L.); 2 Shanxi-Zheda Institute of Advanced Materials and Chemical Engineering, Taiyuan 030024, China; 3 College of Biomedical Engineering, Taiyuan University of Technology, Taiyuan 030024, China; wangyanqin@tyut.edu.cn; 4 College of Architecture and Civil Engineering, Taiyuan University of Technology, Taiyuan 030024, China; luguoyun@tyut.edu.cn (G.L.); gaodongyang0731@link.tyut.edu.cn (D.G.); 5 National Engineering Research Center for Compounding and Modification of Polymer Materials, College of Materials Science and Metallurgy, Guizhou University, Guiyang 550014, China; qinshugao@163.com; 6 Kangmingyuan (Guizhou) Technology Development Co., Ltd., Anshun 561000, China; wanghua@163.com; 7 Ministry of Education Key Laboratory of Macromolecular Synthesis and Functionalization, Department of Polymer Science and Engineering, Zhejiang University, Hangzhou 310027, China

**Keywords:** fly ash, high density polyethylene, composite materials, bellows, mechanical properties, finite element analysis

## Abstract

In recent years, buried bellows have often had safety accidents such as pipeline bursts and ground subsidence due to the lack of adequate mechanical properties and other quality problems. In order to improve the mechanical properties of bellows, fly ash (FA) was used as a reinforced filler in high density polyethylene (HDPE) to develop composites. The FA was surface treated with a silane coupling agent and HDPE-g-maleic anhydride was used as compatibilizer. Dumbbell-shaped samples were prepared via extrusion blending and injection molding. The cross-section morphology, thermal stability and mechanical properties of the composites were studied. It was observed that when 10% modified FA and 5% compatibilizer were added to HDPE, the tensile yield strength and tensile breaking strength of the composites were nearly 30.2% and 40.4% higher than those of pure HDPE, respectively, and the Young’s modulus could reach 1451.07 MPa. In addition, the ring stiffness of the bellows was analyzed using finite element analysis. Compared with a same-diameter bellows fabricated from common commercially available materials, the ring stiffness increased by nearly 23%. The preparation method of FA/HDPE is simple, efficient, and low-cost. It is of great significance for the popularization of high-performance bellows and the high value-added utilization of FA.

## 1. Introduction

There have been problems in urban underground pipe networks for a long time, such as insufficient performance of drainage and sewage pipes, uneven product quality, and so on. It is extremely urgent to improve the mechanical properties of underground pipelines. Nowadays, buried large-diameter double-wall bellows are also gradually being upgraded from cement or steel materials to polymer materials. HDPE has become the preferred material for most bellows manufacturers due to its excellent comprehensive mechanical properties [1]. The introduction of bimodal HDPE further broadened the market of bellows materials. The high molecular weight part yields materials with increased mechanical properties, whereas the low molecular weight part acts as lubricant during HDPE extrusion [2]. Improving the performance of large diameter HDPE bellows is still a difficult problem. Increasing the ring stiffness in HDPE bellows is the key to improving the quality of bellows. On the one hand, the ring stiffness can be increased by improving the mechanical properties of the materials used to prepare the bellows. On the other hand, we can optimize the waveform structure of bellows to improve their performance. In this paper, HDPE composites used for the preparation of bellows were studied, and the ring stiffnesses of bellows with different diameters were calculated using finite element analysis.

With the increasing demand for high-performance bellows and the reduction in the amount of polyethylene raw materials, many people try to add inorganic fillers to HDPE to obtain stronger mechanical properties and cut the use of polyethylene raw materials. There are a large number of studies that report improvements, with an emphasis on the mechanical properties of polyolefins, with the addition of inorganic particles [3]. G. Savini and R. L. Orefice prepared nano-talc/HDPE composites [4]. The results showed that the tensile strength and impact strength of the composites decreased slightly with the addition of talc powder, but the elongation at break increased significantly. M. E. Mahmoud et al. studied the effects of Al_2_O_3_ and BaO nano-additives on the mechanical properties of HDPE [5]. The results indicated that when the content of BaO and Al_2_O_3_ nano-fillers reached 4% and 6% respectively, the increase in Young’s modulus relative to the matrix was 229% and 208% respectively. D. K. Mohan Kumar et al. used HDPE as matrix material and bagasse ash and nano-montmorillonite as reinforcement materials to prepare composites. The results showed that the composites had higher tensile strength and bending strength compared with pure HDPE [6].

As mentioned above, many people add inorganic fillers to polyolefins to enhance the strength or toughness of the materials. However, these inorganic fillers usually come from mining, which easily damages geology and forests. Therefore, many people currently use solid waste as inorganic reinforcing fillers. For example, there are some people who use FA Cenospheres, modified FA, ultrafine FA or other mineral materials as reinforcement materials, which grant HDPE excellent strength and stiffness. FA is an aluminosilicate rich waste with light weight and high strength obtained from coal-fired power plants, whose disposal causes serious environmental pollution [7]. High value-added utilization of FA is an urgent and long-term task [8]. Since polymers are often hydrophobic while FA is hydrophilic, modification of the filler, the matrix, or both is helpful [9]. M. V. Deepthi et al. used a silane coupling agent to treat the surface of FA Cenospheres and prepared FA Cenosphere/HDPE composites. The results indicated that the addition of FA hollow beads could greatly improve the mechanical properties and thermal stability of the composites, but the elongation at break would be reduced [10]. D. Guo et al. studied the effect of ultra-fine FA (UFA) incorporation on the properties of HDPE composites. The results showed that UFA particles without surface treatment could be uniformly dispersed in polyethylene, and UFA had an obvious toughening effect upon rigid particles [11]. However, the addition of UFA greatly reduced the elongation at break of the composite system. In addition, T. T. Wu et al. studied the effect of FA particle size on the mechanical properties of modified FA/HDPE composites [12]. The results showed that the tensile, bending and impact strength of the materials increased at first and then decreased with increasing FA particle size. In the above papers, adding different inorganic fillers to HDPE could improve the strength or toughness of HDPE. However, it was difficult to maintain high strength and toughness at the same time in polyethylene materials. Therefore, it is important to develop a simple, effective and low-cost preparation method for HDPE composite materials.

Pipe circumferential bending stiffness refers to the ability of a pipe to resist circumferential deformation, which is referred to as ring stiffness. Ring stiffness is a very important index for bellows, indicating their ability to resist external pressure [13]. Ring stiffness testing of bellows is generally carried out with a special instrument. Before the measurement, the pipe needs to be prepared and transported to the ring stiffness tester for measurement. This measurement method is not only time-consuming, but also often results in certain measurement errors. In a study by X. P. Wang and Q. Lian, the ring stiffness of bellows was tested using a ring stiffness tester [14]. The results showed that the load, sample length and inner diameter would cause deviation in the measured ring stiffness results. Therefore, the finite element analysis method was used to simulate the ring stiffness testing process in this paper, and the finite element simulation results were then used to verify the potential application value of the prepared HDPE composites in bellows.

In this paper, FA was used as an inorganic filler to enhance the strength and stiffness of HDPE. However, it is usually necessary to add a large volume of filler to the polymer to obtain the desired results [15]. Thus, FA was grafted with a silane coupling agent to obtain treated FA. Next, the reinforced masterbatch was prepared by melt blending a certain amount of FA with LDPE. The blends were then melt blended with HDPE-g-maleic anhydride (HDPE-g-MAH) as compatibilizer. The influence of FA and compatibilizer content on the tensile properties of the material was studied, and the mechanical properties, micro-morphology and thermal stability of the composite materials were analyzed. In addition, the ring stiffness of the bellows prepared by the composite was analyzed using finite element analysis. Based on the analysis and discussion of the above results, the composite material prepared in this paper was not only simple to prepare, low-cost and suitable for the preparation of large-caliber buried bellows, but also contributed to high value-added utilization of FA.

## 2. Materials and Methods

### 2.1. Materials

HDPE (CONTINUUM™ DGDA-2502NT with a melt flow index of 12.4 g/10 min, Dow Chemical Company, Midland, MI, USA);

FA (Shanxi Hujin Coal and Electricity New Materials Company, Taiyuan, China);

3-Amino propyl tri ethoxy silane (APTS) (Nanjing Chuang Shi Chemical Additives Company, Nanjing, China);

Maleic anhydride modified HDPE (HDPE-g-MAH) (FUSABOND™ E265 Functional Polymer, Dow Chemical Company, Midland, MI, USA);

LDPE (LD650 with melt flow index of 22 g/10 min, ExxonMobil, Irving, TX, USA)

Absolute ethanol and glacial acetic acid were obtained from Aladdin Biochemical Technology Co., LTD, (Shanghai, China).

The composition of FA used in this article was provided by Shanxi Hujin Coal and Electricity New Materials Co., Ltd. (Taiyuan, China). The company analyzed the mean composition of the FA using X-ray fluorescence analysis in accordance with the GB/T 14563-2008 test standard in the Table 1.

### 2.2. ATPS-Modified FA

In order to improve the compatibility of the FA and HDPE, the surface of the FA was modified [16]. Silane-modified FA refers to the grafting of APTS onto FA. First of all, anhydrous ethanol and deionized water were fully mixed to produce a mixed solvent. Silane coupling agent (ATPS) was then added and the pH value was adjusted to 5 by adding glacial acetic acid. Next, hydrolyzed ATPS was obtained at room temperature by stirring with a magnetic agitator for 30 min. The mass ratio of the components in the hydrolyzed silane coupling agent was ATPS:deionized water:anhydrous ethanol = 1:1:9. The hydrolyzed ATPS is sprayed on the surface of the FA particles, and its mass fraction is 3% of the FA particles. A high-speed mixer was used to mix the material at high speed for approximately 10 min and then discharge the material. The material was placed in a drying box and dried at 100~120 °C for 2 h to obtain the FA modified by ATPS.

### 2.3. Preparation of FA Masterbatch

In order to distribute FA more evenly within HDPE and prevent it from causing secondary pollution, FA was prepared as a filling masterbatch [17]. A certain amount of LDPE (LD650) and an appropriate amount of polyethylene wax were added to the high-speed mixer and then mixed for 15 min at high speed at a temperature of 80 °C. Then, the same amount of modified FA was added to the high-speed mixer. Run the high-speed mixer first at low speed for 10 min, then at high speed for 10 min, a coarse mixture of FA masterbatch could be obtained. The coarse mixture was added to the extruder for melt extrusion and granulation to obtain the FA masterbatch. Extrusion and granulation were carried out in a micro compounder (HAAKE™ Minilab 3 Micro Compounder, Thermo Scientific, Waltham, MA, USA).

### 2.4. Preparation of the Composites

The particulate composites were obtained by mixing HDPE, the FA masterbatch and the HDPE-g-MAH compatibilizer in various proportions at 210~215 °C. Blending was carried out in a micro compounder (HAAKE™ Minilab 3 Micro Compounder, Thermo Scientific, Dreieich, Germany).

Dumb-bell shaped specimens were then molded as per GB/T 1040.2-2006 specifications, using injection molding (HAAKE™ Mini-Jet Pro, Thermo Scientific, Dreieich, Germany), at 210~215 °C. The amount of compatibilizer was expressed as a weight percentage of FA. The dumb-bell specimens were then subjected to tensile properties and thermal properties testing.

### 2.5. Characterization

Different techniques were used to test the characteristics of the silane-treated FA and HDPE composites in this study. The first technique was scanning electron microscopy (SEM, model JSM-7100F, Tokyo, Japan). For morphology observation, the sample was cryogenically fractured in liquid nitrogen. Samples were then covered with gold and examined with the scanning electron microscope at an operating voltage of 10 kV.

The second technique was thermo-gravimetric analysis (TG-209, Netzsch, Selb, Germany), working at temperatures ranging from 40 °C to 800 °C, a flow rate of 100 mL min^−1^ in a nitrogen atmosphere and a heating rate of 10 °C/min.

The third technique was Fourier transform infrared spectroscopy (FT–IR). FT–IR spectroscopy was performed using a Bruker Tensor 27 FTIR spectrometer (Bruker, Karlsruhe, Germany). All data were recorded at room temperature in the spectral range of 450~4000 cm^−1^. FT–IR spectra were used to compare untreated and treated FA.

A tensile tester (model UTM-4304X, Shenzhen Suns Technology Co., LTD, Shenzhen, China) was used to measure tensile properties. Tensile tests were performed as per GB/T 1040.2-2006. Composite specimens were mounted and subjected to strain at a rate of 5 mm/min until failure occurred. Tensile tests of the composites were carried out at room temperature. Five specimens per batch were tested and the average strength was calculated.

### 2.6. Finite Element Model

#### 2.6.1. Geometry

**Figure 1:**
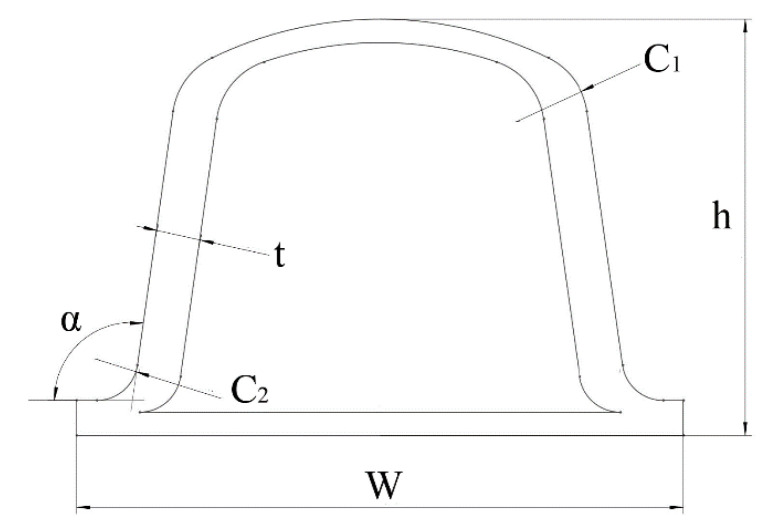
Waveform cross section of a bellows.

The ring stiffness calculation model for the bellows was established using Solidworks software (Version 2021, SolidWorks, Waltham, MA, USA). As shown in Figure 1, the waveform cross sections of different bellows were drawn first. The bellows could be obtained by rotating the corresponding section depending on the different diameter values.

**Figure 2:**
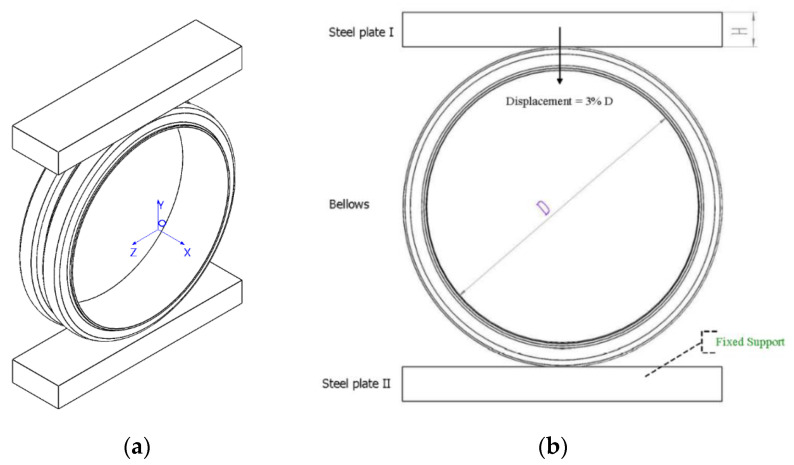
Finite element model of bellows ring stiffness calculation. (**a**) and (**b**) ab are different perspective of the finite element model.

In order to simulate the process of testing the stiffness of the bellows ring, two parallel steel plates were placed at the upper and lower ends of the bellows. The cuboids in Figure 2 were treated as steel plates, which were tangential to the bellows in the x, y and z directions. The length of the cuboids was the sum of the inner diameter of the bellows and the height of the wave crest (D + h), their width was twice that of B, and their height was 50 mm.The ring stiffness of the bellows was tested by compressing the bellows and deforming them by 3%.

#### 2.6.2. Material Parameters

Table 2 shows the material properties of the bellows and steel plates. A FA/HDPE composite material with a Young’s modulus of 1451.07 MPa was selected as the material for the bellows.

#### 2.6.3. Mesh Parameters

In this study, the mesh model was established using Ansys Workbench software (Version 2020 R2, Ansys, Washington, PE, USA). Both the steel plates and bellows adopted the method of automatic meshing, which can be used to automatically select the mesh type for the entity and generate the mesh. Then, the mesh size of the bellows was set. The principle of setting the bellows mesh size was to select the smallest possible mesh unit size that the computer could run.

#### 2.6.4. Boundary and Initial Conditions

The ring stiffness test is described in detail in ISO 9969-94. The basic principle is to place the bellows between two parallel plates and compress it at a constant speed to obtain the curve of the relationship between force and ring stiffness. The ring stiffness is calculated from the reaction force when the pipe produces a deformation of 0.03 times the diameter.

The static structure analysis was performed using the Mechanical APDL solver in Ansys Workbench software (Version 2020 R2). All bellows were subjected to the same boundary conditions and displacement loads. As shown in Figure 2b, a downward displacement load was applied to the lower surface of the upper steel plate, and the displacement was 3%D. The bottom of steel plate was constrained. No load was applied to any part of the bellows.

## 3. Results

### 3.1. FT–IR Spectroscopic Analysis

**Figure 3:**
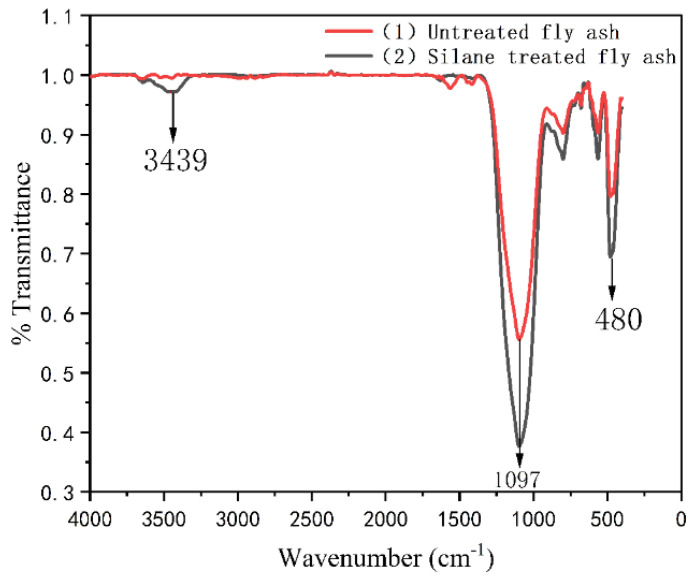
Fourier transform infrared spectroscopy (FT-IR) analysis of silane treated FA and untreated FA.

Figure 3 shows the FT–IR spectra of the untreated and treated FA. The O-Si-O bending vibration is observed at 480 cm^−1^ in Figure 3. It can be observed that the peak strength of FA at 480 cm^−1^ was enhanced after modification. This may be due to the increase in O-Si-O bond content on the surface of the FA or the more stable existence of O-Si-O bonds within FA modified by silane. The absorption peak at 1097 cm^−1^ corresponds to the Si-O single bond’s stretching vibration, which is also enhanced in the spectrum of treated FA. This indicates that many Si-O bonds were added to the surface of the modified FA. This phenomenon is consistent with the results reported by T. T. Wu et al. [12]. In addition, the peak at 3439 cm^−1^ is the stretching vibration region of N-H single bonds. The change in absorption peak strength at 3439 cm^−1^ indicates that the aminopropyl in the silane coupling agent was successfully attached to the surface of the FA.

### 3.2. SEM Analysis of FA

**Figure 4:**
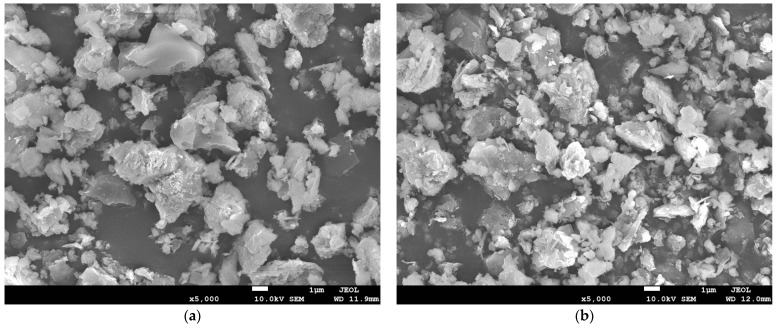
Scanning electron microscopy (SEM) analysis of silane-treated and untreated FA. (**a**) Unmodified FA; (**b**) FA treated with ATPS.

Figure 4a shows the morphological surface of unmodified FA, while Figure 4b is SEM images of dry modified FA. As can be seen from Figure 4a, untreated FA particles were spherical or irregular in shape and the micrograph surfaces were all smooth. Comparing Figure 4b with Figure 4a, it can be seen that the surface of modified FA seemed to be coated with a film and became uneven, which indicates that the surface of FA was successfully modified by ATPS. ATPS was coated on FA through a series of reactions such as hydrolysis, hydrogen bond formation, condensation between silane coupling agent molecules and so on. A similar phenomenon was exhibited by T. T. Wu et al. [12].

### 3.3. Effect of FA on the Tensile Properties of the Composites

**Figure 5:**
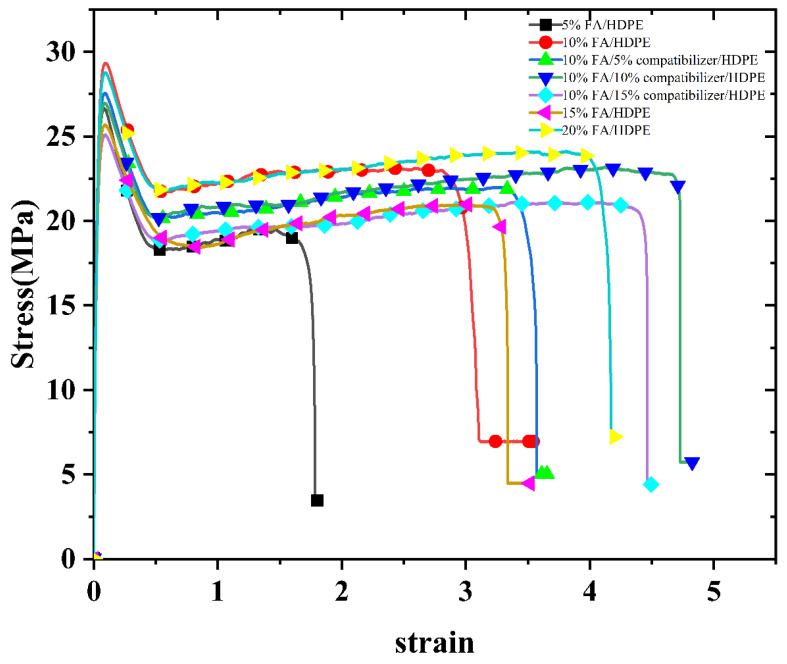
Stress-strain diagrams of different composites.

**Figure 6:**
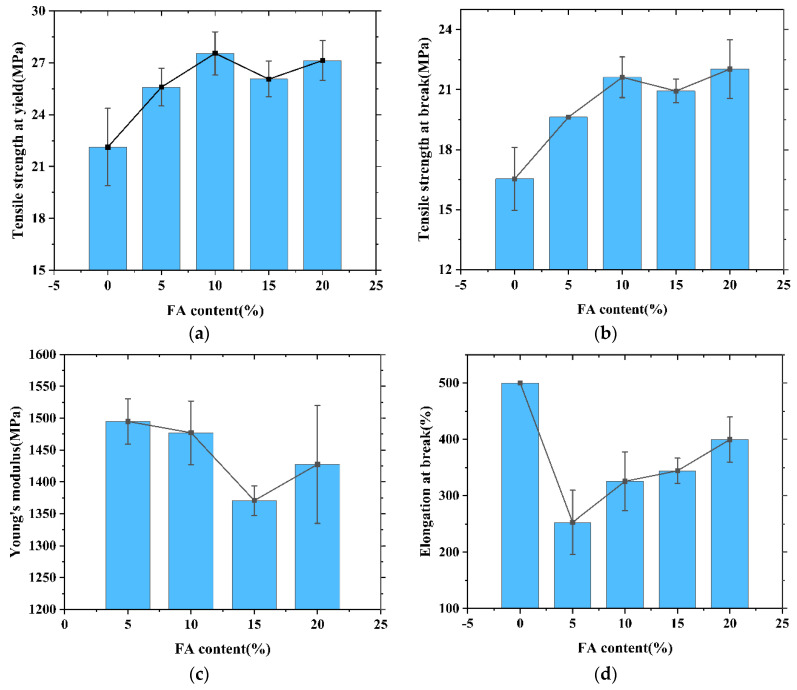
Plot of effect of FA masterbatch on the mechanical properties. (**a**) Tensile strength at yield versus FA masterbatch percentage; (**b**) Tensile strength at break versus FA masterbatch percentage; (**c**) Young’s modulus versus FA masterbatch percentage; (**d**) Elongation at break versus FA masterbatch percentage.

The amount of FA added is one of the important factors affecting the mechanical properties of composites. In this study, FA/HDPE composites were prepared by adding different amounts of FA masterbatch to HDPE. Figure 5 shows the stress–strain curves of different HDPE composites. It can be seen that with the addition of different amounts of FA and compatibilizer, the stress and strain of the composites were similar. The effect of FA on the tensile properties of FA/HDPE composites was studied. The experimental results are shown in Table 3 and Figure 6.

As shown in Figure 6, the tensile yield strength and tensile breaking strength of FA/HDPE composites first increased and then decreased with the increase of FA masterbatch. When the added content of FA masterbatch was 10%, the tensile strength at yield and tensile strength at break reached 27.55 MPa and 21.62 MPa, respectively, which were 24.5% and 30.7% higher than the pure HDPE. This indicates that the addition of a small amount of FA can improve the tensile strength of HDPE. However, when the FA masterbatch content was 15%, the tensile yield strength and tensile breaking strength of the composite materials began to decrease. This may be due to agglomeration of FA particles in HDPE with the increase in FA content, making the HDPE network unable to pack the FA well. This would lead to disconnection in the interface between FA and HDPE when the composite material is subjected to tensile force or bending load. As a result, the crack expansion and stress concentration of the composites will occur, and the tensile strength of the composites will be reduced. When the FA masterbatch content was 20%, the tensile strength was improved.

With increasing addition of FA masterbatch, the change trend of the Young’s modulus of the composites was similar to that of the tensile strength, but the elongation at break of the composites decreased significantly. After adding 10% FA masterbatch, the elongation at break of the composite material significantly decreased from 500% to 325.55%, a decrease of approximately 34.9%. This is because the rigid FA cannot be stretched together with HDPE, so that the elongation at break of the composite material significantly decreased. This phenomenon generally occurs in most composites, where there is always a tradeoff between tensile strength and elongation at break [18]. With further addition of FA masterbatch, the elongation at break of the composite materials was alleviated, which may be due to the toughening effect of LDPE in the FA masterbatch.

### 3.4. Effect of the Compatibilizer on the Properties of the Composites

**Figure 7:**
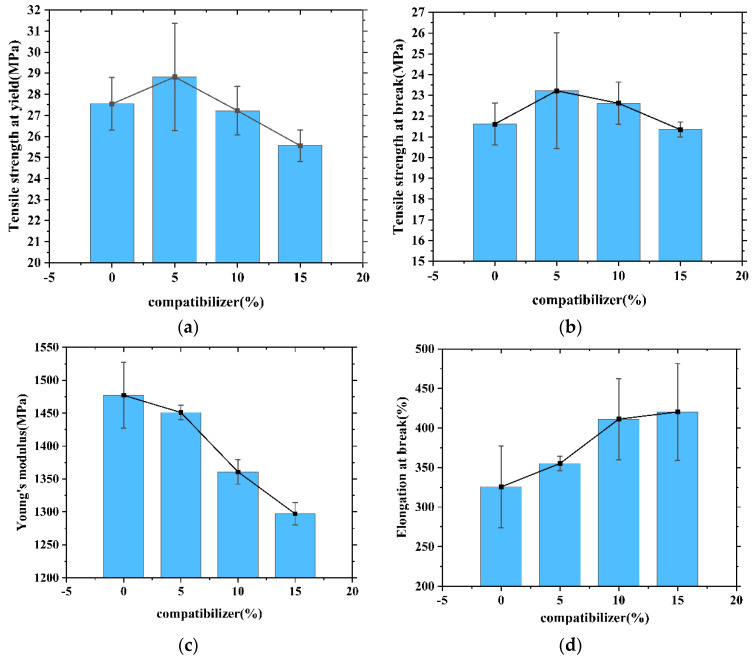
Plot of the effect of compatibilizer percentage on the mechanical properties. (**a**) Tensile strength at yield versus compatibilizer percentage; (**b**) Tensile strength at break versus compatibilizer percentage; (**c**) Young’s modulus versus compatibilizer percentage; (**d**) Elongation at break versus compatibilizer percentage.

It can be seen from Table 3 that the strength and stiffness of the composite reached the highest values when the amount of FA masterbatch was 10%. In order to further improve the mechanical properties of the composites, the effect of compatibilizer addition on the mechanical properties of 10% FA masterbatch/HDPE composites was studied. Table 4 and Figure 7 show the effects of compatibilizer addition on the 10% FA masterbatch/HDPE composite.

According to Figure 7a,b, the tensile strength at yield and tensile strength at break of the 10% FA masterbatch/HDPE composite increased by approximately 5.0% and 7.4% respectively when adding 5% compatibilizer. With an increase in compatibilizer content from 5% to 15%, the tensile strength at yield and tensile strength at break of the composites decreased by approximately 11.1% and 8.1%, respectively.

Figure 7c,d shows the effects of compatibilizer addition on the Young’s modulus and elongation at break of the composite. With the addition of compatibilizer, the Young’s modulus of composite with 10% modified FA masterbatch filling decreased with increasing compatibilizer content, and the elongation at break increased with the addition of compatibilizer. This indicates that the addition of compatibilizer could enhance the strength and fracture toughness of composites and reduce their rigidity.

### 3.5. Composite Morphology

**Figure 8:**
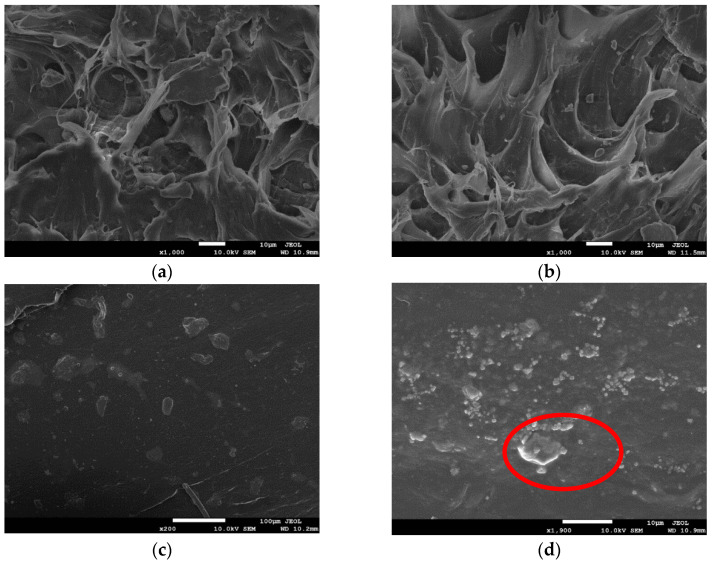
SEM photographs of composites containing: (**a**) HDPE/FA masterbatch (5 wt.%); (**b**) HDPE–FA masterbatch (10 wt.%); (**c**) HDPE–FA masterbatch (10 wt.%) compatibilized with 5% HDPE-g-MAH; (**d**) HDPE–FA masterbatch (15 wt.%).

Figure 8 shows SEM photographs of cross-sections of the composite materials. The liquid nitrogen brittle fracture surfaces of the composite material all presented the phenomenon of ductile fracture. Figure 8a is the SEM image of a cross-section of 5% FA masterbatch/HDPE composite material. It can be seen from Figure 8a that FA did not display a large area agglomeration phenomenon, and HDPE formed a networked structure that wrapped FA particles in it, so the mechanical properties of the composite material were good. Figure 8b is the SEM image of a cross-section of 10% FA masterbatch/HDPE composite material. Compared with Figure 8a, it can be seen that FA was more evenly distributed in HDPE, but a small amount of FA appeared agglomerated and exposed. More rigid FA was encapsulated in HDPE, so the strength and stiffness of the composite material improved. Figure 8c shows the SEM image of a cross-section of the composite material with 10% FA masterbatch, 5% compatibilizer and HDPE. Compared with Figure 8b, the FA was more evenly distributed and was coated by HDPE, and there was no agglomeration of FA, so the mechanical properties of the composite material were better. This was because after adding the compatibilizer, the acid anhydride group in the compatibilizer reacted with the amino group in the modified FA to form chemical bonds, which improved the interaction between FA and HDPE. Because FA could be better encapsulated into high-density polyethylene, the comprehensive mechanical properties of the composites have been further improved. Figure 8d is the SEM image of a cross-section of HDPE composite with 15% FA masterbatch. There was an obvious agglomeration phenomenon of FA, and the resin matrix did not form a networked structure to fully cover the FA, so the strength and stiffness of the composite were reduced.

### 3.6. Thermogravimetric Analysis (TGA)

Table 5 shows the thermogravimetric analysis of pure HDPE and FA/HDPE blends. As can be seen from Table 5, the initial decomposition temperature of the HDPE was 390 °C, and the maximum decomposition temperature was 514 °C, which was caused by -C-C- dissociation in the main chain of HDPE due to the high temperature. Compared to pure HDPE, the 5% FA masterbatch/HDPE composite began thermal degradation at 220.8 °C and reached maximum thermal degradation at 571.75 °C, accompanied by carbon formation of 2.1%. When the content of FA masterbatch was increased to 20% the initial degradation temperature of the composite was 221.93 °C and the thermal degradation tended to be stable at 568.05 °C, and the carbon formation rate was 8.0%. According to the comparison of TG and DTG data of composite materials in Table 5, the initial degradation temperature, fastest degradation temperature and maximum degradation temperature of composite materials were within a small range with increases in the addition of FA, while the carbon formation rate increased gradually with the addition of FA. TGA/DTG analysis also showed that the maximum thermal degradation of all composites was better than that of pure HDPE. The thermogravimetric analysis of composite materials by M. V. Deepthi et al. also obtained similar results [10].

### 3.7. Finite Element Analysis

**Figure 9:**
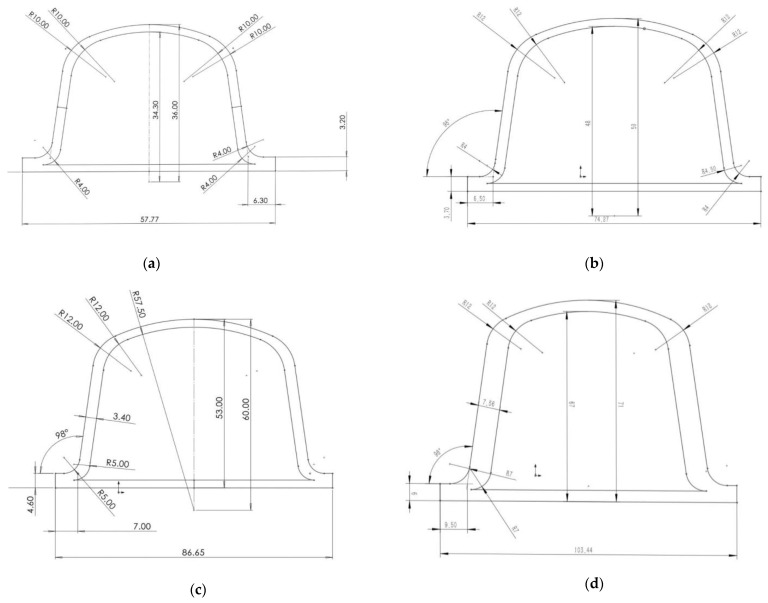

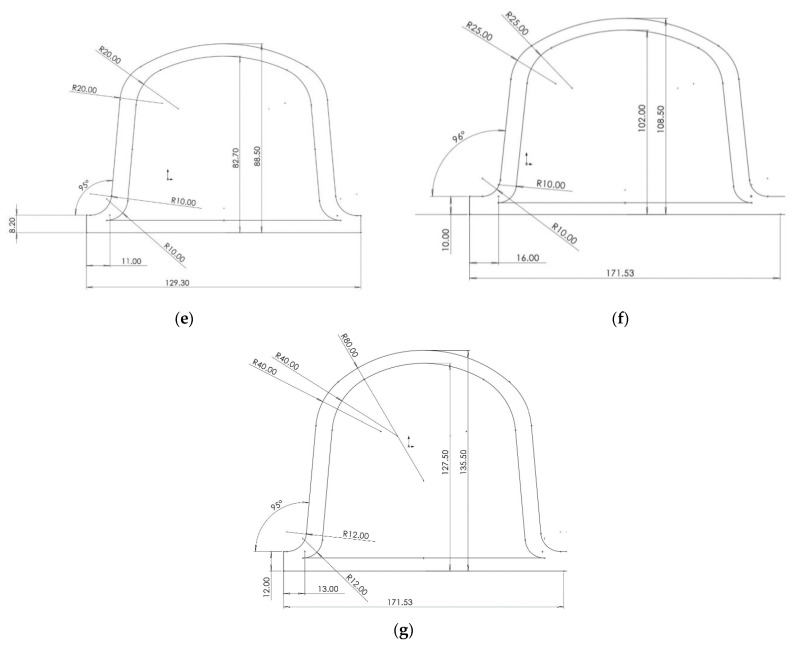
The waveforms of bellows with different diameters as follows: (**a**), 400 mm; (**b**), 500 mm; (**c**), 600 mm; (**d**), 800 mm; (**e**), 1000 mm; (**f**), 1200 mm; (**g**), 1500 mm.

Improving the ring stiffness of large diameter HDPE bellows is a difficult problem, impeding further popularization of the use of large diameter PE pipe. Improving the ring stiffness of large diameter HDPE bellows is key to improving the quality of pipe [19]. In order to verify the performance of a double-wall bellows made from FA/HDPE composites, common large diameter double-wall bellows with diameters of 400~1500 mm were modeled with SOLIDWORKS software. The waveforms of the common diameter bellows are shown in Figure 9.

**Figure 10:**
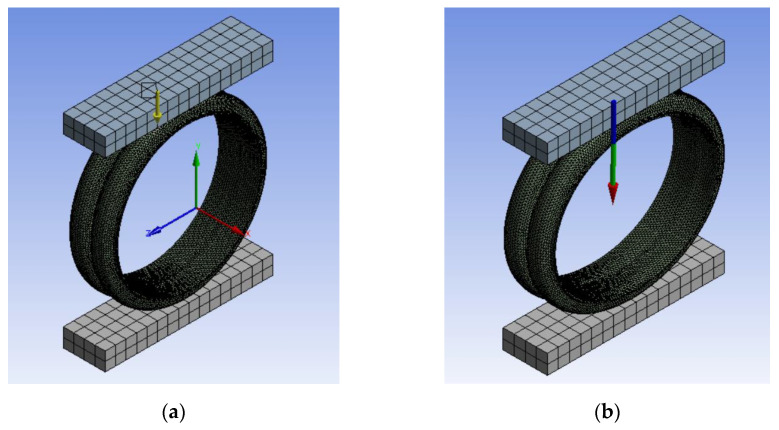
Finite element model of bellows ring stiffness calculation. (**a**) Schematic diagram of steel plate displacement; (**b**) Schematic diagram of reaction force.

Figure 10a is a displacement diagram of the bellows ring stiffness test model. The direction represented by the yellow arrow is the displacement direction of the upper steel plate. Figure 10b is a schematic diagram of the reaction force of the bellows to the steel plate during compression. The reaction force on the upper steel plate could be calculated by Ansys Workbench software (Version 2020 R2, Ansys, Washington, PE, USA). Tthe ring stiffness of the bellows could be calculated by substituting the reaction force into formula (1)

(1) $$S=\left[0.0186+0.025\frac{y}{d}\right]\frac{F}{\mathrm{Ly}}\;$$

where S is the ring stiffness of the pipe; F is the reaction force on the pipe under 3% deformation (KN); L is the length of the specimen (m); y is the amount of deformation (m) of the pipe at 3.0% deformation; d is the diameter of the pipe (m); where y/d = 0.03.

**Figure 11:**
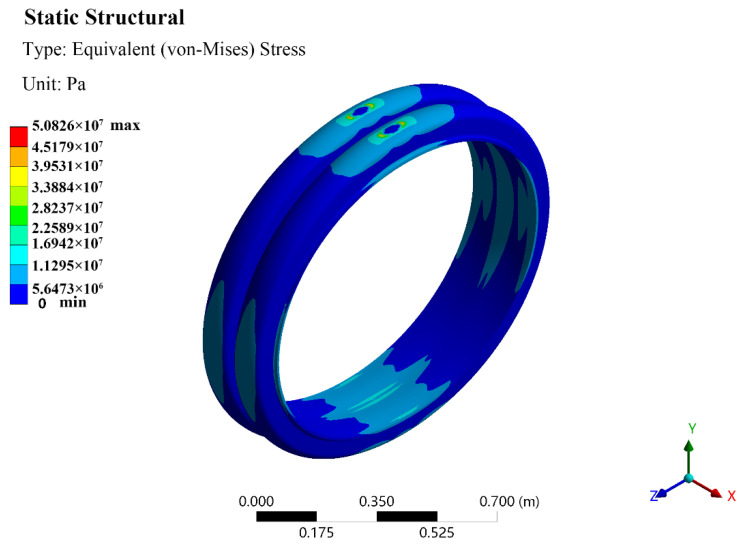
Equivalent stress distribution on a bellows. The diameter of the bellows is 1000 mm.

Figure 11 shows the equivalent stress distribution on a bellows with a diameter of 1000 mm after compression. It can be seen that the stress was mainly concentrated on the upper side of the bellows. The maximum stress occurred on both sides of the tangent part of the upper steel plate. There was no obvious change in the location of the maximum stress upon bellows with different pipe diameters.

**Figure 12:**
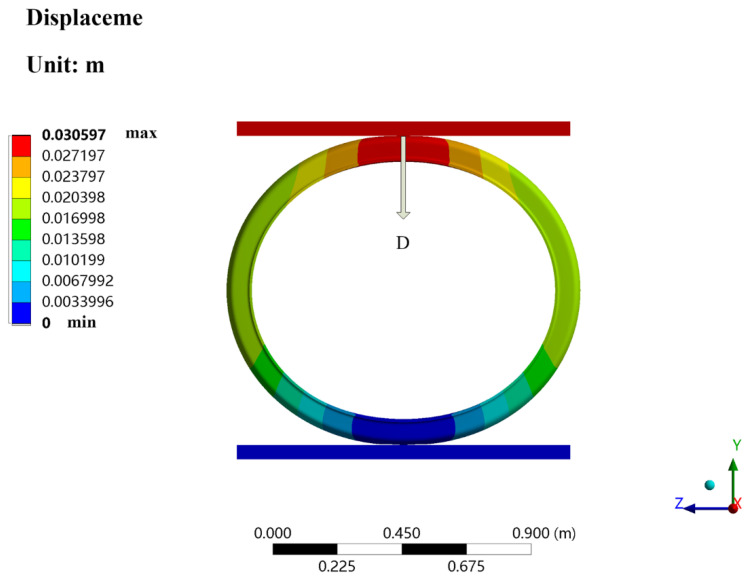
The displacement distribution of a bellows. The diameter of the bellows is 1000 mm.

Figure 12 shows the displacement distribution of the bellows with a diameter of 1000 mm after compression. It is obvious from the figure that the total deformation of the bellows decreased from top to bottom, and the lower steel plate and the part in contact with the lower steel plate were almost not deformed. The deformation mainly occurred above the bellows and focused on the position where it was in contact with the upper steel plate.

Table 6 shows the results of the ring stiffness calculations for the bellows. By comparing these to the ring stiffness of bellows made from common commercially available materials, it was found that the ring stiffness of the bellows increased by approximately 23%, which can better meet the requirements of different use conditions for bellows ring stiffness.

## 4. Discussion

In this paper, high-strength FA was used as an inorganic filler to enhance the mechanical properties of HDPE. When the added quantity of FA masterbatch increased from 0 to 15%, the tensile strength and Young’s modulus of the composites increased at first and then decreased, and the elongation at break decreased continuously with the addition of FA masterbatch, which is consistent with the results of Y. Huang et al. [17]. In contrast to their results, with the further addition of FA masterbatch, the tensile strength, Young’s modulus and elongation at break increased, rather than decreased further. This may be because LDPE in the FA masterbatch further uniformly distributed FA in HDPE.

It is obvious from Figure 5 that the tensile strength of the composites increased at first and then decreased when different amounts of compatibilizers were added to the FA masterbatch/HDPE composites. The improvement in tensile strength in the composites may be attributed to the effective reaction of amino group on the modified FA with the anhydride group in the compatibility agent, which anchored the rigid FA more effectively on the HDPE. Therefore, the stress can be better transferred from the polyethylene matrix to the FA particles. The formation of interactions between filler and matrix materials plays an important role in the improvement of tensile properties [20]. Adding too much compatibilizer reduced the tensile strength of the composites. This may be because the excessive addition of compatibilizer destroyed the highly crystalline structure of HDPE and enhanced the plasticity of the composites, thus reducing the yield strength and breaking strength of the composites. In addition, the addition of compatibilizer increased the elongation at break of the composite. This may be because the addition of the compatibilizer made the FA more evenly distributed in the HDPE matrix, which reduced the occurrence of FA agglomeration and stress concentration within the composite materials.

In a study by M. V. Deepthi et al. [10], HDPE-g-dibutyl maleate was used as a compatibilizer, and the influence of the compatibilizer content on the mechanical properties of HDPE composites was studied. This showed that the tensile strength and Young’s modulus of the composite materials were significantly improved, and the addition of compatibilizers slightly improved the elongation at break of the composite materials. In this study, HDPE-g-MAH, which is relatively easy to buy commercially, was used as a compatibilizer. It can be seen from the above analysis that this method achieved the same effect of strengthening and toughening.

In order to verify whether the ring stiffness of HDPE composites bellows was improved, finite element analysis was used to simulate and calculate the ring stiffness of the bellows. In addition, the ring stiffness of a bellows made from a common commercially available material was also analyzed and calculated, and the ring stiffness of the two was compared. The main tested parameter of the two materials was Young’s modulus. Compared with the ring stiffness of the common material bellows, the ring stiffness of the composite bellows increased by approximately 23%. This shows that the composites are anticipated to be more useful as the preparation material for bellows. The HDPE composites prepared in this paper not only improved the strength and stiffness of the composites, but also alleviated the problem of greatly reduced toughness caused by FA added into HDPE. The utilization of FA not only reduced the preparation cost of large diameter HDPE bellows, but also achieved the reuse of solid waste materials.

At present, common polyethylene bellows usually mix plastic and steel or optimize the wave shape and increase the number of inner ribs to enhance the strength and rigidity of the pipe. The ring stiffness of this bellows was generally 8~12.5 KN/m^2^. The steel plates in the pipe are generally connected by welding, but quality is difficult to guarantee and water leakage is prone to occurring. Due to the complicated preparation process, its production speed is relatively slow. Pure HDPE bellows is also now common and its ring stiffness is generally 4~8 KN/m^2^; its strength has gradually failed to meet increasing needs. In this paper, a high-strength and high-rigidity FA/HDPE composite material was prepared. Compared with the above-mentioned types of bellows, the preparation process was simple and the cost was low. For common bellows diameters, the ring stiffness could reach 10.6~18.4 KN/m^2^. Combining the performance advantages of stiffness and low cost of use, FA/HDPE is a potential high-performance buried bellows material.

## 5. Conclusions

In this study, modified FA was prepared into a masterbatch as a reinforcing filler, and a series of FA/HDPE composite materials were prepared. The influence of the added amounts of FA masterbatch and compatibilizer on the mechanical properties of composite materials was studied. Further studies included cross-sectional scanning electron microscope images and thermal stability of the composite materials. In order to study whether the composite materials were suitable for preparing large-diameter bellows, the ring stiffness of common diameter bellows made of FA/HDPE composites was simulated and calculated using finite element analysis. Based on the experimental results and observations, the following conclusions can be drawn:

(1) The tensile strength and Young’s modulus of the composite increased at first and then decreased with the increase in FA masterbatch content, and the elongation at break decreased with the addition of FA. The existence of LDPE in the FA masterbatch could alleviate the decreases in strength, stiffness and elongation at break caused by excessive addition of FA. When 10% modified FA masterbatch was added to HDPE, the tensile strength of the composite material reached the maximum. Compared with pure HDPE, the tensile yield strength of 10% FA masterbatch/90% HDPE composites was increased by approximately 24.5%, and the tensile breaking strength was increased by approximately 30.7%.
(2) A small amount of compatibilizer could enhance the tensile strength of the composite, but too much compatibilizer would reduce the tensile strength of the composite. The addition of compatibilizer to FA masterbatch/HDPE composite could make FA and HDPE have a stronger interaction. After adding the compatibilizer to the FA masterbatch/HDPE composite materials, the elongation at break of the composite materials would increase, while the rigidity would decrease. When 5% compatibilizer was added to the 10% FA masterbatch/HDPE composite, the comprehensive mechanical properties of the composite were the best. Compared with the 10% FA masterbatch/HDPE composite, the tensile yield strength, tensile breaking strength and elongation at break of the composite increased by 4.6%, 7.4% and 9.1%, respectively.
(3) The ring stiffness of bellows made of 10% modified FA masterbatch/5% HDPE-MAH/85% HDPE composite was calculated by finite element analysis, and it was found that the bellows made of this composite could achieve higher ring stiffness. Compared with the common diameter bellows made from common commercially available materials, the ring stiffness increased by approximately 23%. Therefore, this composite is expected to be a material for the preparation of large diameter HDPE bellows.

## Figures and Tables

**Table 1:** Mean composition of fly ash.

Composition	Mass Fraction	Statistical Error
Carbon	2.72	2
Moisture	0.87	2.8
SiO_2_	46.1	0.2
Al_2_O_3_	40.5	0.18
CaO	2.58	0.47
TiO_2_	1.91	0.52
SO_3_	1.85	0.75
Fe_2_O_3_	1.65	0.25
K_2_O	0.58	0.96
MgO	0.56	1.79
Na_2_O	0.12	3.01
BaO	0.07	7.14
ZrO_2_	0.06	4.58
SrO	0.06	0.44
CeO_2_	0.05	0.51
V_2_O_5_	0.03	5.34
Chlorine	0.01	9.15

**Table 2:** Material parameter.

Parameter	Structural Steel	Material a ^1^	Material b ^2^
Density	7850 kg/m^3^	970 kg/m^3^	970 kg/m^3^
Young’s modulus	2 × 10^5^ MPa	1451.1 MPa	1181.0 MPa
Poisson ratio	0.3	0.38	0.38

^1^ Material a: self-made composite material; ^2^ Material b: a common commercially available material with Young’s modulus of 1181.41 MPa.

**Table 3:** Effect of FA on tensile properties of composites.

Blend Composition	Tensile Strength at Yield	Tensile Strength at Break	Young’s Modulus	Elongation at Break
Pure HDPE	22.13 ± 2.23 MPa	16.54 ± 1.57 MPa		500.00% ± 0%
5% FA masterbatch/95% HDPE	25.60 ± 1.09 MPa	19.63 ± 0.01 MPa	1495.0 ± 35.4 MPa	252.85% ± 56.56%
10%FA masterbatch/90% HDPE	27.55 ± 1.25 MPa	21.62 ± 1.01 MPa	1477.2 ± 49.9 MPa	325.55% ± 51.97%
15%FA masterbatch/85% HDPE	26.06 ± 1.03 MPa	20.93 ± 0.60 MPa	1370.1 ± 23.2 MPa	344.20% ± 22.56%
20%FA masterbatch/80% HDPE	27.13 ± 1.15 MPa	22.03 ± 1.47 MPa	1327.5 ± 92.3 MPa	399.61% ± 40.10%

**Table 4:** Effects of compatibilizer addition on the tensile properties of the composites.

Blend Composition	Tensile Strength at Yield	Tensile Strength at Break	Young’s Modulus	Elongation at Break
10% FA masterbatch/90% HDPE	27.55 ± 1.25 MPa	21.62 ± 1.01 MPa	1477.2 ± 49.9 MPa	325.55% ± 51.97%
10% FA masterbatch/5% compatibilizer/85% HDPE	28.82 ± 2.54 MPa	23.23 ± 2.80 MPa	1451.1 ± 10.8 MPa	355.15% ± 9.05%
10% FA masterbatch/10% compatibilizer/80% HDPE	27.22 ± 1.16 MPa	22.62 ± 1.01 MPa	1360.8 ± 18.8 MPa	411.28% ± 51.33%
10%FA masterbatch/15% compatibilizer/75% HDPE	25.56 ± 0.74 MPa	21.35 ± 0.36 MPa	1297.1 ± 17.1 MPa	420.33% ± 61.47%

**Table 5:** Thermogravimetric Analysis of FA/HDPE Composites.

Blend Composition	Initial Temperature	Fastest Temperature	Peak	Initial Temperature
Pure HDPE	390.00 °C	465.00 °C	514.00 °C	0%
95%HDPE/5%FA masterbatch	220.80 °C	431.34 °C	571.75 °C	2.1%
90%HDPE/10%FA masterbatch	239.58 °C	432.18 °C	569.60 °C	4.6%
85%HDPE/15%FA masterbatch	229.13 °C	439.55 °C	590.46 °C	7.1%
80%HDPE/20%FA masterbatch	221.93 °C	430.74 °C	568.05 °C	8.0%

**Table 6:** Comparison of ring stiffness of bellows with different pipe diameters.

Bellows Diameter	S_1_	S_2_	Percentage Increase
DN400	11.5 KN/m^2^	9.4 KN/m^2^	22.9%
DN500	10.6 KN/m^2^	8.7 KN/m^2^	22.9%
DN600	11.8 KN/m^2^	9.6 KN/m^2^	22.8%
DN800	18.7 KN/m^2^	15.2 KN/m^2^	22.9%
DN1000	18.4 KN/m^2^	15.0 KN/m^2^	22.8%
DN1200	16.9 KN/m^2^	13.7 KN/m^2^	22.9%
DN1500	18.2 KN/m^2^	14.8 KN/m^2^	22.9%

S_1_: Ring stiffness of bellows self-made from composite material; S_2_: Ring stiffness of bellows made from a common commercially available material with Young’s modulus of 1181.41 MPa.

## Data Availability

The data that support the findings of this work are available from the corresponding author on reasonable request.
